# Hepatic epithelioid hemangioendothelioma: a report from three university centers*

**DOI:** 10.1590/0100-3984.2015.0059

**Published:** 2016

**Authors:** Antonello Giardino, Frank H. Miller, Bobby Kalb, Miguel Ramalho, Diego R. Martin, Karina Rodacki, John T. Woosley, Richard C. Semelka

**Affiliations:** 1 MD, Department of Radiology, University of North Carolina at Chapel Hill, Chapel Hill, NC, USA; Universitá degli Studi di Milano, IRCCS Policlinico San Donato, San Donato Milanese, Milan, Italy.; 2 MD, FACR, Department of Radiology, Northwestern Memorial Hospital, Northwestern University Feinberg School of Medicine, Chicago, IL, USA.; 3 MD, Department of Radiology, University of Arizona Medical Center, Tucson, Arizona, USA; Department of Radiology, The Emory Clinic, Wesley Woods Geriatric Hospital, Atlanta, GA, USA.; 4 Department of Radiology, University of North Carolina at Chapel Hill, Chapel Hill, NC, USA; Department of Radiology, Hospital Garcia de Orta, Almada, Portugal.; 5 MD, PhD, Department of Radiology, University of Arizona Medical Center, Tucson, Arizona, USA; Department of Radiology, The Emory Clinic, Wesley Woods Geriatric Hospital, Atlanta, GA, USA.; 6 MD, Department of Radiology, University of North Carolina at Chapel Hill, Chapel Hill, NC, USA; Ecomax - Centro de Diagnóstico por Imagem, Blumenau, SC, Brazil.; 7 MD, PhD, Department of Pathology & Laboratory Medicine, University of North Carolina at Chapel Hill, Chapel Hill, NC, USA.; 8 MD, Department of Radiology, University of North Carolina at Chapel Hill, Chapel Hill, NC, USA.

**Keywords:** Liver neoplasms/diagnosis, Epithelioid hemangioendothelioma, Magnetic resonance imaging

## Abstract

**Objective:**

To determine common imaging findings of hepatic epithelioid hemangioendothelioma on magnetic resonance images.

**Materials and Methods:**

A search was made of three institutional databases between January 2000 and August 2012. Seven patients (mean age, 47 years; range, 21-66 years; 6 women) with pathology-confirmed diagnosis of hepatic epithelioid hemangioendothelioma who had undergone magnetic resonance imaging were identified. None of the patients had received any treatment for hepatic epithelioid hemangioendothelioma at the time of the initial magnetic resonance imaging examination.

**Results:**

Hepatic epithelioid hemangioendothelioma tumors appeared as focal masses in 7/7 patients, greater than 5 in number, with a coalescing lesion in 1/5, and peripheral localization in 6/7. Capsular retraction was present in 4/7, and was associated with peripherally located lesions. Early ring enhancement was appreciated in the majority of lesions in 7/7 patients. Centripetal progressive enhancement was shown in 5/7 patients on venous phase that exhibited a distinctive thick inner border of low signal on venous phase images, and a central core of delayed enhancement. Small lesions did not show this.

**Conclusion:**

The combination of multifocal round-configuration lesions that are predominantly peripheral and exhibit early peripheral ring enhancement and late appearance of an inner thick border of low signal and central core of high signal may represent an important feature for hepatic epithelioid hemangioendothelioma.

## Abstract (pt)

**Objetivo:**

Determinar os achados comuns de hemangioendotelioma epitelioide hepático em imagens de ressonância magnética.

**Materiais e Métodos:**

Uma pesquisa foi feita em três bases de dados institucionais entre janeiro de 2000 e agosto de 2012. Sete pacientes (média de idade, 47 anos; variação, 21-66 anos; 6 mulheres) com diagnóstico confirmado por exame patológico de hemangioendotelioma epitelioide hepático submetidos a ressonância magnética foram identificados. Nenhum dos pacientes havia recebido tratamento para hemangioendotelioma epitelioide hepático antes do exame inicial por ressonância magnética.

**Resultados:**

Tumores de hemangioendothelioma epitelioide hepático apareceram como massas focais, maiores que 5 em número, em 7/7 pacientes, com uma lesão coalescente em 1/5 e localização periférica em 6/7 pacientes. Retração capsular esteve presente em 4/7 pacientes e foi associada com lesões perifericamente localizadas. Realce precoce em anel foi visto na maioria das lesões, em 7/7 pacientes. Realce progressivo centrípeto foi demonstrado em 5/7 pacientes na fase venosa, que exibia uma borda interna espessa distinta de baixo sinal nas imagens de fase venosa e um núcleo central de realce tardio. As lesões pequenas não mostraram isso.

**Conclusão:**

A combinação de lesões de configuração arredondada multifocais que são predominantemente periféricas e exibem realce precoce em anel periférico e aparecimento tardio de uma borda espessa interna de baixo sinal e um núcleo central de alto sinal pode representar uma característica importante para hemangioendotelioma epitelioide hepático.

## INTRODUCTION

Hepatic epithelioid hemangioendothelioma (HEHE) is a rare vascular neoplasm (incidence 1/1,000,000) of endothelial origin that may arise in various soft tissues and visceral organs^(1,2)^. It was first described in 1982 by Weiss et al.^(2)^, and as a primary hepatic occurrence in 1984 by Ishak et al.^(3)^. HEHE is known to occur in individuals of all ages (reported from 3 to 86 years) with a peak incidence in the third and fourth decades of life and a greater frequency in women than men (3:2)^(4,5)^. Etiologic factors are currently unknown and although several risk factors have been proposed, none has been proven to increase the risk of developing HEHE, including hepatitis virus and chronic liver disease^(4)^.

About half of patients with HEHE present with right upper quadrant discomfort, hepatomegaly, and/or weight loss, and approximately one quarter are asymptomatic^(4)^. Normal serum alpha-fetoprotein, carcinoembryonic antigen, and cancer antigen 19-9 are typical lab values of patients with HEHE^(4)^, and 15% of HEHE patients have normal liver function tests values^(4)^. Extrahepatic involvement at the time of diagnosis was observed by Mehrabi et al. in 36.6% of patients, with lung, peritoneum, lymph nodes, and bone being the most common sites^(4)^. Liver transplantation is currently the most common and preferred treatment^(4,6,7)^ with 5-year survival rates ranging from 64% to 83%^(8,10)^, and limited extrahepatic disease should not be considered an absolute contraindication to liver transplantation^(4)^.

Prior computed tomography (CT) and magnetic resonance imaging (MRI) studies have reported features of HEHE including ring enhancement^(11,14)^. Due to the finding of rim enhancement of HEHE lesions after contrast administration, it is not uncommon to mischaracterize HEHE on radiological imaging as metastatic disease. To our knowledge, a more in-depth analysis of these lesions has not been performed in order to determine if imaging features were present that were distinctive for HEHE, which in part reflects the rarity of this lesion, and therefore the lack of studying sufficient numbers to identify findings.

The purpose of our study was to determine common imaging findings of HEHE on MRI images based on the experience of three university centers.

## MATERIALS AND METHODS

### Patients

A computerized search in the radiological database reports of the three institutions was performed using the keywords "epithelioid hemangioendothelioma", for consecutive patients between January 2000 and August 2012, who had abdominal MRI examinations. In a separate part of the study, a search in the pathology department records for pathologically proven cases of HEHE was also made for the involved institutions and within the same time period. This information was then cross-referenced to find all patients with pathologically proven HEHE and MRI. Seven patients (mean age, 47 years; range, 21-66 years; 6 women) with a pathologyconfirmed diagnosis of HEHE who had undergone MRI were identified. Clinical history of each patient was obtained from the institutional computer information system. Three patients with a pathological record of HEHE but without MRI were not eligible. The patients' data are displayed in Table 1.

**Table 1:** Patient characteristics and clinical data.

Patient	Age (years)	Gender	Hepatitis B virus / Hepatitis C virus	Serum alphafetoprotein (ng/mL)	Alkaline phosphatase	CD31 / CD34 endothelial cell markers	Pathological sample
1	21	Female	Not available / Not available	84	76	Positive / Positive	Core biopsy
2	29	Female	Negative / Negative	2	88	Positive / Positive	Wedge resection + core biopsy
3	51	Male	Negative / Negative	< 5	462	Positive / Positive	Open liver biopsy
4	64	Female	Not available / Not available	Not available	102	Positive / Positive	Core biopsy
5	55	Female	Not available / Not available	3	149	Not available / Not available	Core biopsy
6	42	Female	Negative / Negative	3	149	Not available / Not available	Core biopsy
7	66	Female	Not available / Not available	Not available	—	Not available / Not available	Surgical resection

The primary indications for imaging were: abdominal pain (3/7); abdominal distension (1/7); increasingly fatigue (1/7); a cecal mass seen at colonoscopy (1/7); and cholecystitis (1/7). None of the patients had received any treatment for HEHE at the time of the initial MRI examination hence the imaging features were reflective of the natural state of the lesions.

### Pathological analysis

Lesions were confirmed histopathologically (see Table 1 for more details regarding the tissue specimens) as nests or cords of epithelioid endothelial cells spreading within sinusoids and other vascular structures in a background of highly myxoid to hyaline stroma, and intense staining with CD31 (platelet endothelial cell adhesion molecule 1) and CD34 (human hematopoietic progenitor cell antigen) and factor VIII at immunohistochemistry, which confirms the endothelial origin of the tumor cells according to the tumor classification of the World Health Organization^(4,15)^. Biopsies were performed after the MRI study.

### MRI technique

Six of seven MRI examinations were performed at 1.5 T (Vision, Symphony, Sonata or Avanto; Siemens Medical System, Malvern, PA, USA) and one at 3 T (Trio; Siemens Medical Systems) MRI systems, using a phased-array torso coil. In all patients, standard upper abdomen protocol, including pregadolinium and postgadolinium sequences, was performed. Gadobenate dimeglumine (MultiHance; Bracco Diagnostics, Milan, Italy) was administered in six of seven patients and gadoxetate disodium (Eovist; Bayer Schering Pharma AG, Berlin, Germany) in one; they were administered by a power injection (Medrad, Pittsburgh, PA, USA) as a bolus of 0.05-0.1 mmol/kg gadolinium chelate at 2 mL/s. Postcontrast sequences were acquired at approximately 18 s (hepatic arterial dominant phase), 45-60 s (venous phase), and 90-120 s (interstitial phase) after gadolinium administration. Technical parameters used in the 1.5 T system were as follows: 2D gradient-echo pre and postcontrast, axial plane, inphase and out-of-phase, TR 140-200 ms, TE 4.4 ms/2.4 ms, flip angle 80°, slice thickness 8 mm, matrix size 128 × 256, and acquisition time 20 s (two patients); and 3D gradientecho pre and postcontrast, axial plane, fat saturation, TR 4.3 ms, TE 1.7 ms, flip angle 10°, slice thickness 3.5 mm, matrix size 144 × 320, and acquisition time 19 s (two patients). One patient was examined in the 3 T system, with the following parameters: 3D gradient-echo pre and postcontrast, axial plane, fat saturation, TR 3.07 ms, TE 1.32 ms, flip angle 13°, slice thickness 3 mm, matrix size 256 × 256, and acquisition time 19 s.

### Image analysis

MR images were retrospectively reviewed on a picture archiving and communication system (PACS, Impax; Agfa-Gevaert, Mortsel, Belgium) by two radiologists in consensus. They had four years of experience in body MRI. They were not aware of specific clinical and pathologic findings, but with knowledge of the diagnosis of HEHE.

The following characteristics were evaluated: tumor morphology (focal or coalesced), localization (peripheral or central) and presence of capsular retraction; tumor contours (regular or irregular) and margins (well- or ill-defined); signal intensity on unenhanced T1- and T2-weighted MR images (mildly, moderately, or highly hypointense/hyperintense); lesional pattern of signal was identified on post gadolinium T1-weighted images.

## RESULTS

No patient had a clinical history of chronic liver disease. Serum alpha-fetoprotein and alkaline phosphatase tests were available on the computer information system in four and five patients, respectively. One of seven patients had a significantly elevated alpha-fetoprotein and another one had an abnormal alkaline phosphatase (Table 1). Five of seven patients were positive at immunostaining for both CD31 and CD34 endothelial markers (they were not available in two patients).

**Figure 1:**
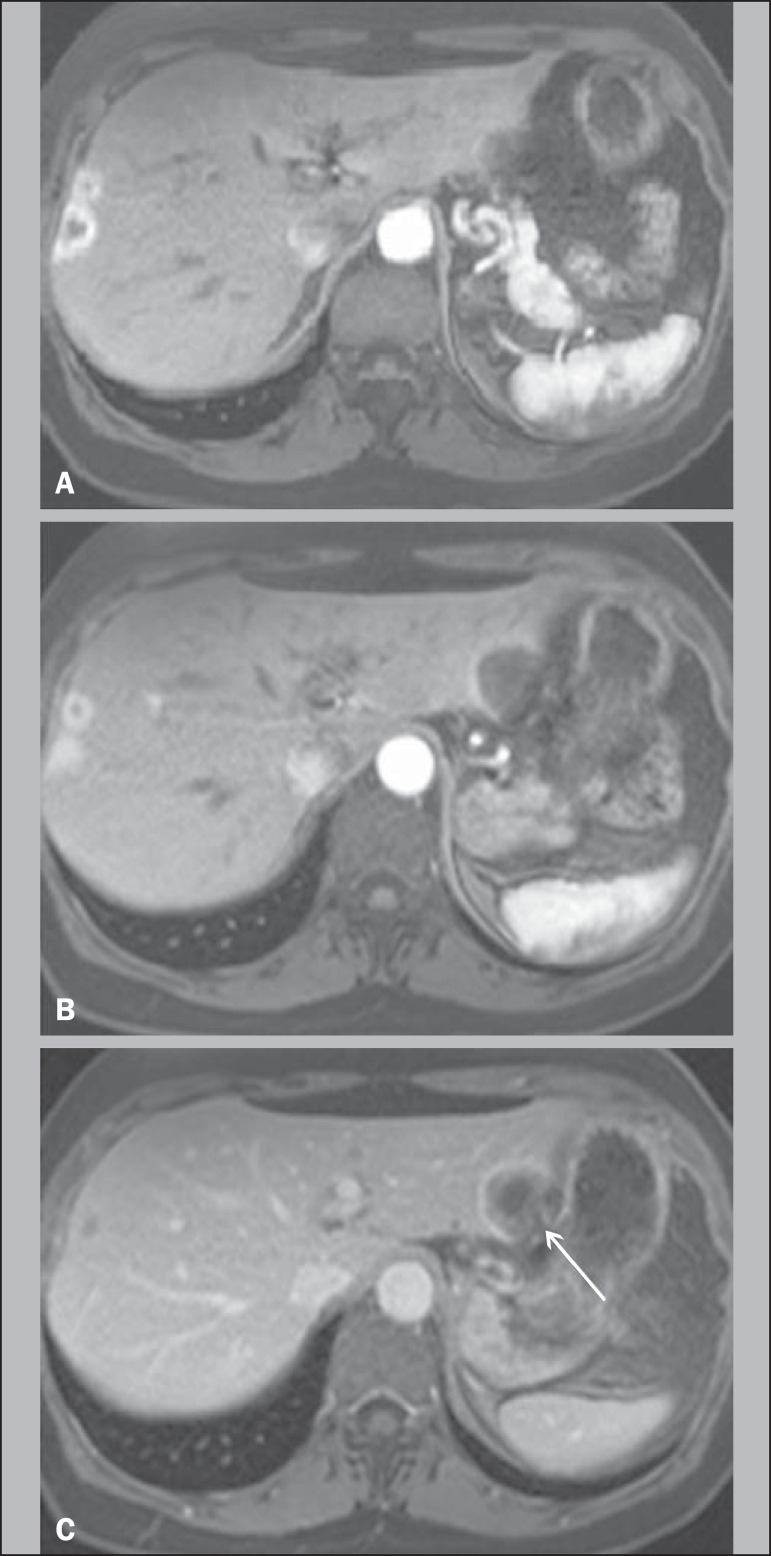
A 64-year-old female with HEHE. Axial, postcontrast T1-weighted fatsuppressed 3D MR images at 1.5 T. Small lesions in the right lobe show thick rim of enhancement on hepatic arterial dominant phase (**A**). A large subcapsular lesion in the left lobe show thin rim of enhancement in the same phase (**B**). The latter lesion, imaged on venous phase, showed capsular retraction (arrow, **C**).

**Figure 2:**
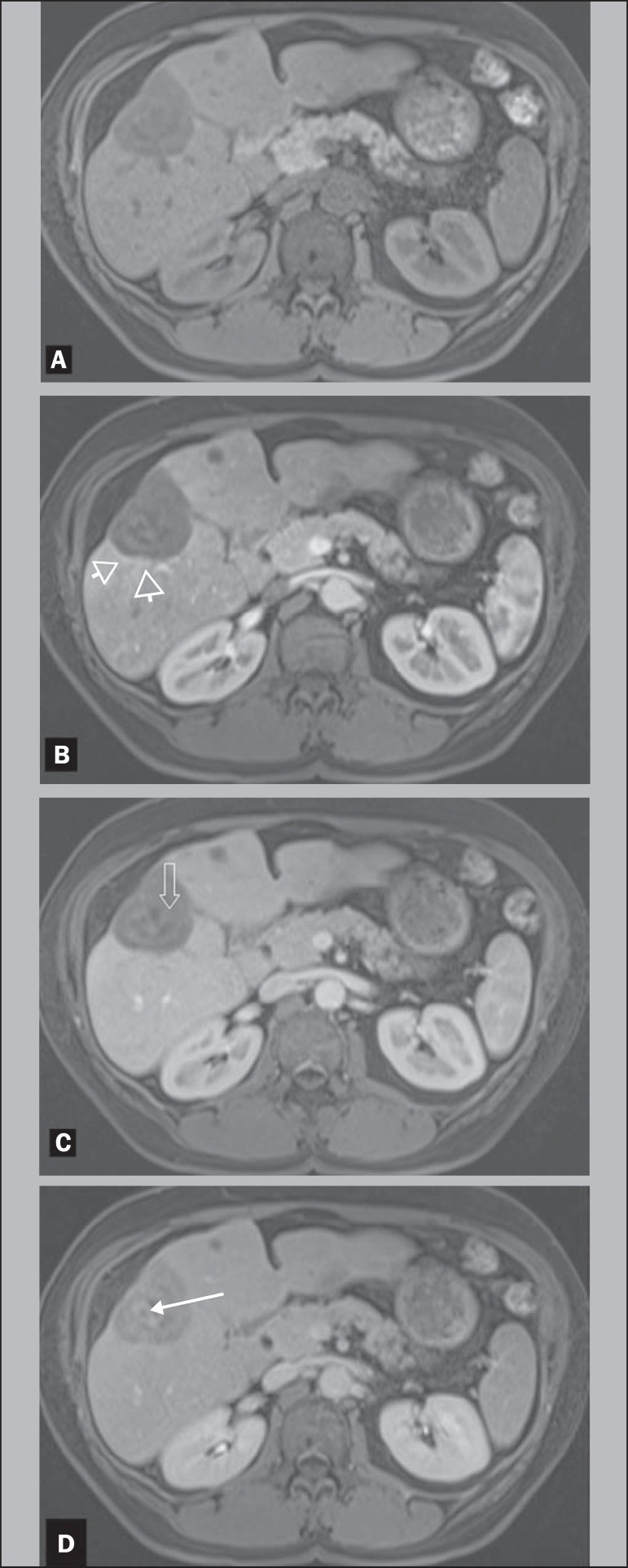
A 42-year-old female with HEHE. Axial, T1-weighted pre and postcontrast MR images at 1.5 T. The same lesion in the right lobe of the liver, showing marked hepatic volume loss due to capsular retraction. Well demonstrated is again the characteristic pattern of enhancement, with a thin rim of enhancement on hepatic dominant arterial phase (arrowheads, **B**), thick inner border of low signal on venous phase (arrow, **C**) and central core of delayed enhancement on interstitial phase (arrow, **D**). Noted is also a small lesion in segment IV.

**Figure 3:**
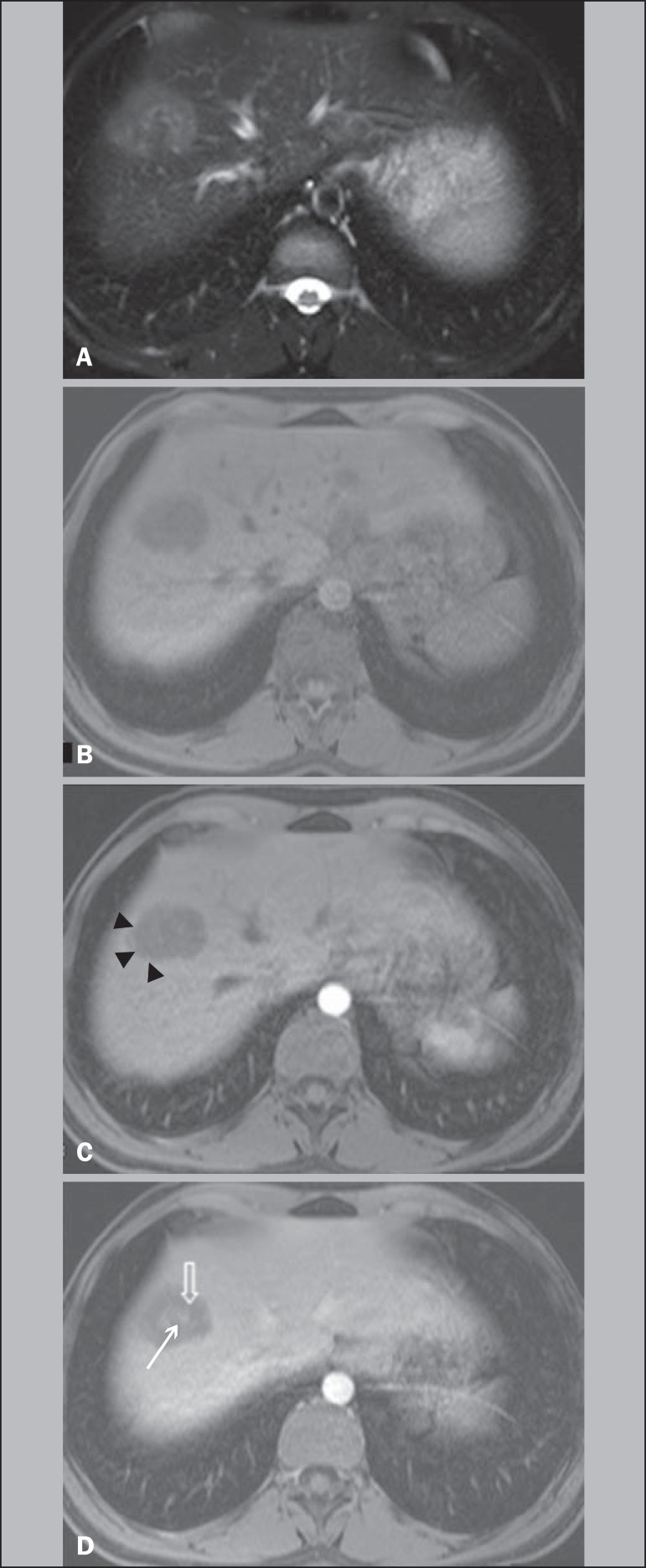
A 21-year-old female with HEHE. MR images at 1.5 T. Axial T2-weighted fat-suppressed (**A**), pre (**B**) and postcontrast T1-weighted fat-suppressed 3D images in the hepatic arterial dominant phase and venous phase (**C,D**). A subcapsular lesion is shown in the hepatic dome that appears to be heterogeneously moderate hyperintense on T2-weighted image (**A**). On postcontrast imaging, there is a thin rim-enhancement on hepatic arterial dominant phase (arrowheads, **C**), centripetal progression with a thick inner border of low signal and a central core of late enhancement on venous phase (open arrow and thin arrow, respectively, D). No evidence of hepatic capsular retraction.

The MRI findings of the seven patients with HEHE are displayed in Table 2. The HEHE tumors appeared as focal masses, greater than 5 in number, in 7/7 patients, and peripheral localization in 6/7. One of seven had concurrent peripheral and central lesions, and a coalesced lesion was present in this patient, and was peripheral. This patient showed several cystic appearing lesions and the majority of the liver parenchyma was replaced by tumor lesions. Capsular retraction was present in 4/7, and was associated with peripherally located lesions (Figures 1 and 2). All patients demonstrated lesions with a heterogeneous mild to moderate hyperintensity on T2-weighted images (Figure 3) and a homogeneous moderate hypointensity on T1-weighted images (Figure 2). The lesions possessed rounded configuration, except for the one infiltrative lesion. Ring enhancement was appreciated in the majority of lesions in 7/7 patients on hepatic arterial dominant phase. This ring pattern was a thin rim (2-3 mm) of peripheral enhancement in 3/7 cases, and a thick rim (7-8 mm) in one patient with only < 1.5 cm lesions (Figures 1 and 2). Three patients with multiple lesions, ranging from < 1.5 cm to > 2 cm, showed both thick rim and thin rim patterns of ring enhancement, respectively. Centripetal progressive enhancement was shown in 5/7 patients on venous phase, that exhibited a thick inner border of low signal on venous phase images, which appeared as low signal on all phases of enhancement (Figures 1, 2 and 3). A central core of delayed enhancement was observed in all lesions > 2 cm (Figures1 and 3) in 5/7 patients. The small lesions did not show this (Figure 1).

**Table 2:** MRI findings.

Patient	Pattern	Localization	Contours	Margins	T1W	T2W	HADP	Venous	Interst	Caps Retr
1	MF	P	Reg	Well def	Mod hypo	Mild hyper	Thin rim	TIB	CCo	No
2	MF	P	Reg	Well def	Mod hypo	Mod hyper	Thick rim	Thin rim	CentP	No
3	MF + Coa	P + C	Reg	Well def	Mod hypo	Mod hyper*	Thin rim	Thin rim	CentP	No
4	MF	P	Reg	Well def	Mod hypo^†^	Mild hyper^‡^	Thin /Thick rim	Thin rim / TIB	CCo / CentP	Yes
5	MF	P	Reg	Well def	Mod hypo	Mild hyper	Thin rim	TIB	CCo	Yes
6	MF	P	Reg	Well def	Mod hypo	Mod hyper	Thin / Thick rim	Thin rim / TIB	CCo / CentP	Yes
7	MF	P	Reg	Well def	Mod hypo	Mild hyper	Thin / Thick rim	Thin rim / TIB	CCo / CentP	Yes

T1W, T1-weighted images; T2W, T2-weighted images; HADP, hepatic arterial dominant phase; Venous, venous phase; Interst, interstitial phase; Caps Retr, hepatic capsular retraction; MF, multifocal; MF + Coa, multifocal + coalescing; P, peripheral; P + C, peripheral + central; Reg, regular; Well def, well defined; Mod hypo, moderate hypointense; Mild hyper, mild hyperintense; Mod hyper, moderate hyperintense; TIB, thick inner border of low signal; CCo, central core of late enhancement; CentP, centripetal progression of enhancement.

* Some lesions had cystic appearance.

† One lesion had calcified rim.

‡ One lesion had cystic appearance.

## DISCUSSION

HEHE is classified as a malignant neoplasm by the World Health Organization^(15)^, and the majority (>85%) of patients present with multifocal, bilobar lesions on radiological imaging, yet the clinical course of HEHE is variable, and intermediate in prognostic seriousness in the spectrum of vascular tumors between benign hemangiomas and malignant angiosarcomas, with reports of patient deaths ranging from within weeks of diagnosis to living up to 27 years without treatment^(4,5,16)^. Orthotopic liver transplantation is currently considered the treatment of choice^(17)^, with demonstration of long-term survival even in the presence of distal metastases. Transcatheter arterial chemoembolization has been shown to be valuable when extrahepatic disease or comorbid conditions prohibit transplantation. Hence, based on its relatively favorable course, distinguishing HEHE from other hepatic tumors is crucial^(18)^.

The pathologist's awareness is essential because of the variable patterns of the tumor, which may mimic other lesions^(19)^. In fact, Makhlouf et al. reported that approximately 60% to 80% of patients with HEHE were initially misdiagnosed as cholangiocarcinoma, angiosarcoma, hepatocellular carcinoma (HCC), metastatic carcinoma, or sclerosing hemangioma on histopathology^(5)^.

HEHE nodules are composed of a fibrous myxoid or hyalinized stroma with a relatively hypocellular center with fibrous septa causing capsular flattening and retraction as they progress^(20)^. The tumor margins show increased cellularity with active proliferation of dendritic and epithelioid cells. Demonstration of the vascular or endothelial origin of the tumor is critical for diagnosis and requires immunostaining for endothelial markers, including CD31, CD34 and factor VIII-related antigen.

On radiological imaging, two different patterns of HEHE are described: a multifocal nodular type, hypothesized to be an early stage of the disease, and a diffuse type, which is thought to be an advanced stage, where the nodules have grown and coalesced to form large confluent masses^(21)^. HEHE are usually peripheral in location, and confluent masses are almost invariably seen in the periphery of the liver, owing to the extension of the tumor through the tributaries of the portal and hepatic veins.

The actively proliferating peripheral margins of HEHE lesions, shown at histopathology likely correlate with the finding of a peripheral rim enhancement on hepatic arterial dominant phase images that may resemble metastatic carcinomas. Ring enhancement was appreciated in the majority of lesions in 7/7 patients on hepatic arterial dominant phase images. The ring enhancement was a thin rim of peripheral enhancement in 3/7 cases, and was a thick rim in one case, with this latter case representing small, < 1.5 cm, lesions. Three patients showed both thin rim and thick rim of enhancement on hepatic arterial dominant phase, respectively on > 2 cm and < 1.5 cm lesions (Figure 2).

The hypocellular portion with fibrous septa of HEHE lesions on histopathology may correlate with the findings of a hypovascular inner border (Figures 1 and 3), as observed in 5/7 patients. A centripetal gradual progression of contrast enhancement was present in all cases, with five patients showing late appearance of central core enhancement (Figures 1 and 3). This combination of thin peripheral enhancement, inner border of low signal and central core enhancement confers a multilayered appearance. This has been previously described as "target" or "halo" sign^(11,14)^.

Comparing HEHE to metastases, both lesions may be numerous and show rounded configuration when > 2 cm in size (with the notable exception of colon cancer metastases which exhibit a cauliflower type margination). Central enhancement and peripheral washout, described for metastases, does resemble the thick inner border of low signal and central core on late enhancement images. This appearance for metastases may only be observed in hypervascular metastases, typically gastrinoma^(22)^. The important difference is that the inner border of low signal in HEHE never exhibited intense enhancement on hepatic arterial dominant phase images, and therefore this appearance did not represent a wash-out phenomenon, but rather a persistently hypoenhancing tumor zone. As with metastases that show late central enhancement, the late central core enhancement in HEHE may reflect diffusion of contrast into the central matrix of the tumor with delayed venous withdrawal. Histopathologic evaluation of the vascularity and interstitial spaces of HEHE may explain the mechanisms for the persistently hypovascular inner border and late enhancing central core.

Comparing HEHE with hemangiomas, both exhibit peripheral enhancement. Hemangiomas characteristically possess nodular discontinuous ring on hepatic arterial dominant phase images, which is different than the thin rim of HEHE. HEHE lesions are more numerous than hemangiomas, although hemangiomas commonly are multiple, but 2-3 in number. HEHE also maintained a rounded configuration when they were large in diameter (except when lesions coalesced), whereas hemagiomas typically develop a lobular border when they are larger than 5 cm. Both HEHE and hemangiomas show centripetal enhancement reflecting the primary vascular nature of these tumors; different from the centripetal progression of homogeneous enhancement of hemangiomas, HEHE showed a persistent inner border of low signal. Enhancement of a central core on delayed images was a common features of HEHE > 2 cm, whereas in hemangiomas > 5 cm almost invariably show lack of central enhancement.

The distinction from HCC is straightforward as the great majority of HCCs show washout and late capsule enhancement on delayed images, and hepatic arterial phase ring enhancement is very rare. Furthermore HCCs are most often observed in individuals with chronic liver disease/cirrhosis.

Cholangiocarcinomas and mixed HCC-cholangiocarcinomas tend to show early diffuse heterogeneous enhancement with retention of contrast on delayed images, but a clear definition of an inner border of hypoenhancement and a core of central enhancement has not been described for these lesions. Cholagiocarcinomas also rarely are multifocal to the extent we have observed for HEHE.

A peripheral location in the hepatic parenchyma with retraction or flattening of the underlying liver capsule has been described as an important feature for HEHE^(21,23)^. In the current study, we found a focal pattern in all seven patients, with a peripheral localization in 6 and both peripheral and central lesions in one; this latter patient had also a peripheral coalescing lesion. Capsular retraction was present in 4/7 patients, and was associated with more peripherally located lesions (Figures 2 and 3). No capsular retraction was observed in three patients. One of them had only lesions < 2 cm in size, that might be the explanation in that case, but one patient had only lesions > 3 cm, and the other one had multiple small lesions and one large (> 5 cm) subcapsular lesion, hence this feature may be present in more than half of individuals with this entity. Capsule retraction overlying the tumor is more commonly associated with intrahepatic cholangiocarcinomas^(21,24,25)^, where this features has been combined secondary to portal vein compression and secondary hepatic parenchymal atrophy. Capsule retraction is seen in treated hepatic metastases, and in cases of confluent fibrosis in cirrhosis^(21,24,25)^, where retraction from fibrosis is the responsible feature.

Additional aspects of HEHE, is that the tumor was mainly observed in young females. This has also been described in other series^(12,14)^. No patient had a history of malignancy, nor chronic liver disease. No biliary dilatation was observed with these lesions.

On MRI the HEHE nodules have been reported to have moderate to high inhomogeneous signal intensity on T2-weighted images, and low signal intensity on T1-weighted images^(21,23,26)^. All of our patients demonstrated lesions with heterogeneous mild to moderate hyperintensity on T2-weighted images (Figure 1) and a homogeneous moderate hypointensity on T1-weighted images (Figure 3). These findings are relatively nonspecific. However, inhomogeneity on T2-weighted images may reflect lower signal intensity of fibrotic, necrotic, or hemorrhagic areas, and higher signal intensity zones of vascular proliferation and edematous connective tissue^(23)^. Calcifications are also described in 20% of patients, as reported by Makhlouf et al.^(5)^, and we observed a calcified-rim lesion in 1/7 patient, however MRI is relatively insensitive to the detection of calcifications.

As with metastases and HCC there may be an evolution in the appearance of lesions of HEHE. The small lesions showed relatively thick ring enhancement with homogeneous central progression of enhancement (Figure 2). The thicker peripheral rim may be a feature of the small size of the lesions, reflecting greater biological activity as a function of greater vascularization.

In this case these lesions are difficult to distinguish from metastases. It has also been previously proposed that as tumor grows, the associated fibrosis becomes sufficiently dense and sclerotic to cut off the circulation to the neoplastic cells^(5)^, and this may account for both the inner border of low signal and the late central core enhancement, showed by larger lesions. The inner border of low signal representing tissue, with sparse vascularization and the late central core, representing tissue in which contrast has diffused in but has remained relatively trapped by the lack of adequate venous drainage.

The other individual with an atypical appearance for HEHE had lesions located both peripherally and centrally to occupy the majority of the liver parenchyma, several of the lesions with a cystic appearance. This may represent an appearance of advanced mature disease of HEHE, reflecting progressive devascularization.

Our study has some limitations. This study was retrospective. However, the most important limitation of our study was the small number of patients, which reflects the rarity of HEHE. We attempted to compensate for this by including data from three centers with busy MRI practices.

In summary, the combination of multifocal round-configuration lesions that are predominantly peripheral and exhibit early thin rim enhancement and late appearance of an inner thick border of low signal and central core of high signal may represent a distinctive feature for HEHE.
